# Mouse knockout models for HIV-1 restriction factors

**DOI:** 10.1007/s00018-014-1646-8

**Published:** 2014-05-23

**Authors:** Jan Rehwinkel

**Affiliations:** Medical Research Council Human Immunology Unit, Medical Research Council Weatherall Institute of Molecular Medicine, Radcliffe Department of Medicine, John Radcliffe Hospital, Headley Way, University of Oxford, Oxford, OX3 9DS UK

**Keywords:** Restriction factor, HIV-1, SAMHD1, APOBEC3, Tetherin

## Abstract

Infection of cells with human immunodeficiency virus 1 (HIV-1) is controlled by restriction factors, host proteins that counteract a variety of steps in the life cycle of this lentivirus. These include SAMHD1, APOBEC3G and tetherin, which block reverse transcription, hypermutate viral DNA and prevent progeny virus release, respectively. These and other HIV-1 restriction factors are conserved and have clear orthologues in the mouse. This review summarises studies in knockout mice lacking HIV-1 restriction factors. In vivo experiments in such animals have not only validated in vitro data obtained from cultured cells, but have also revealed new findings about the biology of these proteins. Indeed, genetic ablation of HIV-1 restriction factors in the mouse has provided evidence that restriction factors control retroviruses and other viruses in vivo and has led to new insights into the mechanisms by which these proteins counteract infection. For example, in vivo experiments in knockout mice demonstrate that virus control exerted by restriction factors can shape adaptive immune responses. Moreover, the availability of animals lacking restriction factors opens the possibility to study the function of these proteins in other contexts such as autoimmunity and cancer. Further in vivo studies of more recently identified HIV-1 restriction factors in gene targeted mice are, therefore, justified.

## Introduction

Viruses infect organisms from all domains of life. The evolutionary pressure they exert on their hosts is evident from the presence of a multitude of defence mechanisms. Examples for strategies of how different organisms ensure immunity to virus infection include the CRISPR system in bacteria [1] or RNA interference in plants and invertebrates [2, 3]. Perhaps not surprisingly, viruses evade and counteract such antiviral mechanisms, and this in turn results in the emergence of new host defence pathways. Indeed, higher organisms typically employ a variety of measures to contain and eliminate infecting viruses. For example, in mammals, cytotoxic T-cells, antibodies, natural killer cells, interferons and a variety of antiviral proteins all contribute to the immune response that ensues following virus infection.

This review focuses on virus restriction factors. Most definitions of the term agree that a restriction factor is a host protein that directly inhibits a stage in the life cycle of a virus in a cell-intrinsic manner. In other words, restriction factors need to be present in an infected cell and typically counteract infection in that cell or in some cases are transferred to the next target cell. Here, I will use this definition, although additional criteria are sometimes applied [4, 5]. Indeed, restriction factors are often under positive selection and are typically targeted by viral antagonists, reflecting the evolutionary arms race between virus and host. Further, the expression of mammalian restriction factors is often induced by cytokines such as the type I interferons, important mediators of antiviral immunity. Some restriction factors are highly specific and control only a narrow range of viruses, whereas other restriction factors broadly target several classes of viruses. Proteins such as pattern-recognition receptors that are involved in detecting the presence of viruses and in inducing an antiviral state are typically not considered to be restriction factors. Instead, restriction factors are characterised by direct modes of action against the life cycle of a virus. In principle, the concept of restriction factors is applicable to any virus and host. However, the term was initially coined and is still most commonly used in the context of mammalian retrovirus infection.

### The importance of studying restriction factors in vivo

Restriction factors are often studied in experiments using cultured cells. Their overexpression is predicted to inhibit virus replication, while their loss-of-function is presumed to have the opposite outcome enhancing virus replication. Indeed, such effects are typically taken as evidence that a protein is a virus restriction factor. Biochemical and structural analyses often provide mechanistic insight into how a restriction factor antagonises virus infection and pinpoint the step in the virus life cycle that is targeted. Collectively, data from these types of in vitro studies are immensely valuable in identifying and characterising proteins that can restrict viruses. Moreover, the mechanistic data generated by in vitro studies often suggest new avenues for therapeutic antiviral interventions.

A full understanding of the biology of restriction factors, however, can only be obtained if in vitro data are complemented by in vivo experiments, testing the role of a restriction factor in a living organism. Indeed, how restriction factors contribute to immunity of the host to virus infection can only be investigated in vivo, given the complexity of the antiviral immune response. Small animal models can be used as a tool to dissect the contribution of restriction factors to antiviral immunity in vivo, and several restriction factors have been genetically ablated in the mouse. Virus challenge experiments in such knockout mice have begun to reveal how restriction factors contribute to the control of viruses at an organismal/systemic level and have provided a number of surprising insights into the biology of restriction factors.

### Restriction factors targeting retroviruses

Retroviruses are a large family of diverse viruses. Sub-families include the beta- and gamma-retroviruses, typified by murine mammary tumour virus (MMTV) and murine leukaemia virus (MLV), respectively, and the more complex lentiviruses such as human immunodeficiency virus 1 (HIV-1). Retrovirus particles are enveloped by a lipid bilayer membrane derived from host cells and contain an RNA genome. Viral proteins present in the virion include reverse transcriptase, integrase, structural proteins forming a capsid around the RNA genome and transmembrane glycoproteins. The latter mediate attachment to target cells and facilitate entry via fusion of the viral envelope with cellular membranes. As a result, the viral RNA genome and associated proteins enter the cytosol of infected cells. In addition to viral proteins, some cellular proteins such as APOBEC3G (see below) can also be packaged into virions and delivered into new target cells. Next, reverse transcriptase converts the RNA genome into complementary DNA (cDNA), which is then integrated into the genome of the infected cell. Lentiviruses transport the viral pre-integration complex, containing cDNA and integrase, across nuclear pores and are, therefore, able to infect quiescent, non-dividing cells. In contrast, many other retroviruses depend on the breakdown of the nuclear envelope during mitosis to gain access to the host genome and only infect dividing cells. The next step in the life cycle of retroviruses is transcription of the integrated proviral DNA into viral messenger RNA. Viral proteins are translated and assemble new virions together with two copies of the RNA genome transcribed from the provirus. Last, progeny virus particles bud from the cell membrane, which in some cases is followed by a maturation step of the virion.

**Fig. 1:**
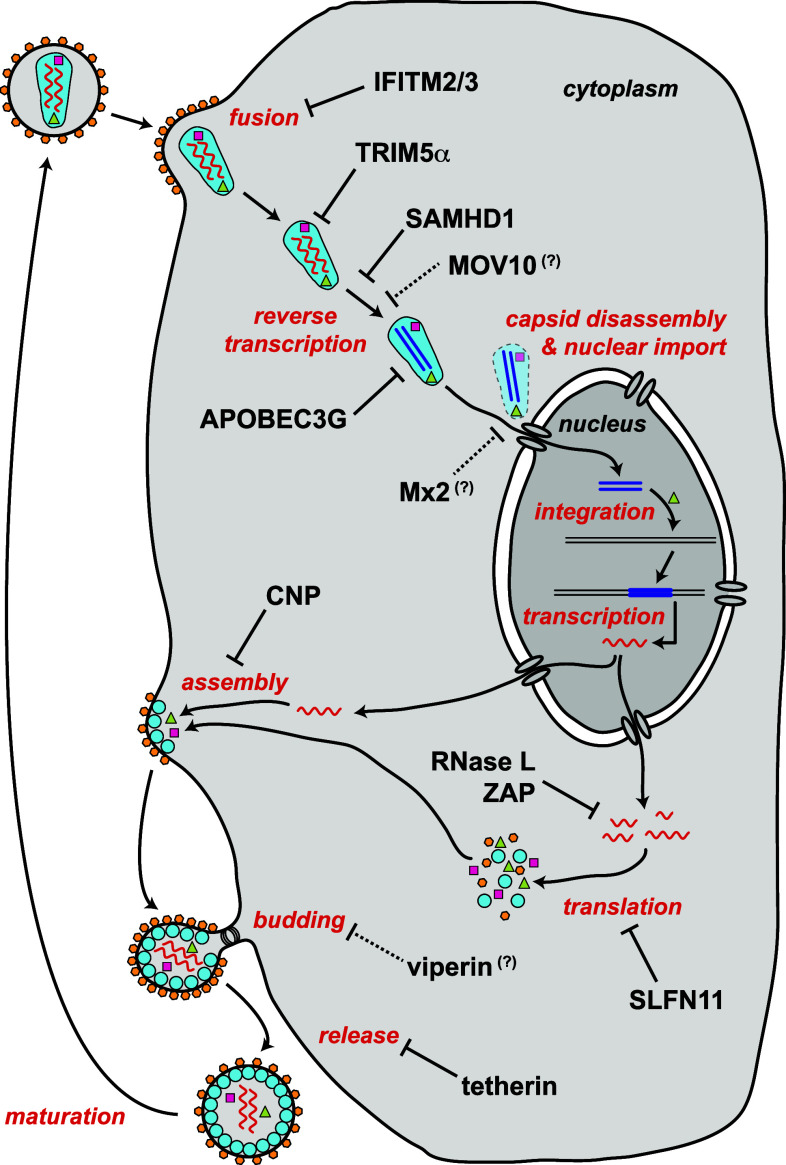
HIV-1 restriction factors. The function of selected HIV-1 restriction factors is shown in the context of the HIV-1 life cycle. Restriction factors are indicated in *black* and steps in the viral life cycle are in *red*. Viral proteins are represented by *coloured symbols*, including reverse transcriptase (*violet squares*), integrase (*green triangles*) and envelope glycoprotein (*orange hexagons*). Viral RNA is represented by a *wavy red line* and viral DNA by a *straight dark blue line*. The functions of MOV10, Mx2 and viperin are speculative as indicated by *question marks and dotted arrows*. Please refer to the text for details

Many of these steps of the retroviral life cycle are targeted by restriction factors. Figure 1 summarises some of the currently known HIV-1 restriction factors in the context of the infection cycle. In the next sections, selected HIV-1 restriction factors are discussed and data from mouse knockout models are summarised. Mice are not the natural hosts of HIV-1 and a number of blocks to infection exist in rodent cells [6]. Nevertheless, several HIV-1 restriction factors are conserved between human and mouse (Table 1). Moreover, some aspects of the HIV-1 life cycle can be studied in murine models, for example by circumventing a block to entry by pseudotyping viral particles with vesicular stomatitis virus glycoprotein (VSV-G). It is, therefore, possible to characterise at least some of the functions of these proteins during HIV-1 infection by genetic ablation in the mouse. In addition, mice are naturally infected by a number of beta- and gamma-retroviruses, and in vivo infections of knockout mice with these viruses have provided new insights into the biology of restriction factors, some of which can perhaps be extrapolated to HIV-1.

**Table 1:** Selected HIV-1 restriction factors discussed in this review

Human HIV-1 restriction factor	Step in the viral life cycle or viral molecule targeted	Mouse orthologue	Knockout mouse models	In vivo retrovirus infections
IFITM1-3	Fusion and possibly other targets	Mouse IFITM1-3 are not direct orthologues of human IFITM1-3 [16]	IFITM1: [197]	Not tested
			IFITM2: Not available	
			IFITM3: [198, 199]	Not tested
CNP	Assembly	1-to-1 (*)	[200]	Not tested
Viperin	Egress and possibly other targets	1-to-1 (*)	[32, 33]	Not tested
Tetherin	Release	1-to-1 (*)	[40, 41]	In vivo restriction of MLV (LP-BM5) and, if induced by type I interferon, M-MLV
SAMHD1	Reverse transcription	1-to-1 (*)	[76, 77]	Controls VSV-G pseudotyped HIV-1 derived vectors, but not M-MLV or Friend virus in short-term experiments
APOBEC3G	cDNA integrity and reverse transcription	APOBEC3	[132, 134, 138, 139]	Restricts MMTV, M-MLV, Friend virus and MLV (LP-BM5) in vivo
TRIM5α	Capsid	No clear 1-to-1 mouse orthologue		
MOV10	Reverse transcription and possibly other targets	1-to-1 (*)	Not available	
Mx2	Late post-entry	*Mx2* contains a frameshift mutation in many inbred laboratory strains [177]	Not applicable	
RNase L	Viral RNA	1-to-1 (*)	[201]	No effect on Friend virus levels or induction of adaptive immune responses
ZAP	Targets viral transcripts for degradation	1-to-1 (*)	[187]	Not tested
SLFN11	Translation of viral transcripts	No clear 1-to-1 mouse orthologue		

* See http://www.ensembl.org/

It is noteworthy that the very concept of virus restriction is based on observations made in murine models of retrovirus infection [7, 8]. Work carried out almost 50 years ago discovered that certain inbred strains of mice are sensitive to infection with Friend virus, whereas others are not [9, 10]. Friend virus is a mix of a replication-competent helper virus and a replication-defective transforming virus and is a model for gamma-retrovirus infection. Friend virus causes erythroleukaemia in susceptible strains of mice. Crosses between resistant and susceptible strains revealed that resistance is inherited in a dominant way. Mapping of the genes underlying the resistance phenotype led to the discovery of some of the first restriction factors [11, 12]. One of these, encoded by the *Fv4* gene, restricts MLV infection by a mechanism called receptor interference. The Fv4 protein blocks access of the envelope glycoprotein of the infecting virus to its cognate receptor [7, 13]. *Fv4* is derived from an endogenous retrovirus. It is related to the MLV envelope glycoprotein, but—due to mutations—Fv4 is non-functional as a viral envelope glycoprotein. Nevertheless, it is still able to interact with and mask cellular receptors required for MLV uptake, thereby preventing infection with exogenous virus. Another restriction factor uncovered by these experiments in mice is encoded by the *Fv1* gene. As for *Fv4*, the *Fv1* gene is derived from an endogenous retrovirus. It encodes a protein related to the capsid protein of exogenous retroviruses [14]. Indeed, the *Fv1* gene product targets the capsids of incoming viruses and appears to interfere with their subcellular trafficking [14].

These studies of retrovirus restriction in mice paved the way for the discovery of many more restriction factors controlling a variety of viruses, including some important human pathogens. This review discusses selected restriction factors that counteract HIV-1, with a focus on what has been learned from recent mouse knockout models.

## Restriction of HIV-1 at the membrane

Fusion of the viral envelope to cellular membranes as well as budding of progeny virus particles from the plasma membrane are key steps in the retroviral life cycle. Both are targeted by restriction factors.

As discussed earlier, the *Fv4* gene controls MLV infection in mice by blocking the interaction of the virus with its receptor on host cells [7, 13]. Whether lentiviruses such HIV-1 are restricted by a similar pathway—i.e. expression of an envelope-mimic from an endogenous retrovirus—is unknown. Nevertheless, recent data suggest that entry of HIV-1 is indeed inhibited by host factors, namely the interferon inducible transmembrane (IFITM) proteins. The IFITMs are a family of small proteins with two transmembrane domains and are involved in cell adhesion, cell proliferation, development, bone formation and host–pathogen interactions [15–18]. The expression of three IFITM proteins, IFITM1-3, is induced by type I interferons, suggestive of an antiviral function. Indeed, RNA interference screening identified a role for IFITM1-3 in controlling RNA virus infection [19]. An overexpression screen of interferon-stimulated genes revealed that IFITM3 and perhaps IFITM2 can control HIV-1 [20]. Further in vitro studies using overexpression of IFITM1-3 and depletion of these proteins by RNA interference substantiated the evidence that these proteins are HIV-1 restriction factors [21–23]. Consistent with the notion that IFITM3 restricts RNA virus infection by blocking fusion [24, 25], it is believed that IFITM3 and IFITM2 proteins also inhibit fusion of HIV-1 [21, 22]. It is possible that the role of IFITM proteins in controlling HIV-1 depends on cell type and that these proteins restrict the virus by multiple mechanisms [18, 22, 23]. For example, IFITM1 has been suggested to restrict HIV-1 after entry [22]. These are important questions for future work and it will also be interesting to challenge mice lacking IFITM proteins with retroviruses to determine the in vivo relevance of these factors to anti-retroviral defence. Whether retroviruses encode IFITM antagonists is another interesting issue for future studies.

Not only entry of retroviruses into host cells but also their egress following successful genome replication is controlled by restriction factors. One example is 2′,3′-cyclic-nucleotide 3′-phosphodiesterase (CNP) that has recently been shown to be involved in counteracting assembly of progeny virions. CNP is associated with the cell membrane, is encoded by an interferon stimulated gene and inhibits a late stage in assembly of HIV-1 progeny virions [26]. Mechanistically, it has been proposed that CNP blocks assembly after the structural protein Gag and viral RNA have associated with the plasma membrane and that it interacts with Gag [26]. Mouse CNP is unable to control HIV-1 particle production in overexpression settings [26]. It will be interesting to determine if mouse CNP controls MLV and other mouse retroviruses, and to determine if and how it shapes virus infections in vivo.

Viperin (also known as CIG5 and RSAD2) is another protein that may restrict egress of HIV-1. Viperin is a transmembrane protein encoded by an interferon stimulated gene and localises to the endoplasmic reticulum [27, 28]. An antiviral function against a variety of viruses has been demonstrated, but the mechanisms by which Viperin exerts these effects are not fully understood [27, 28]. One possible antiviral mechanism by which viperin may control budding of enveloped viruses is modification of the lipid environment within the cell [27, 28]. Viperin has been proposed to control HIV-1 in vitro [29–31], although some of the inhibitory effect might be virus strain and/or cell type specific [29]. Viperin-deficient mice are available [32, 33], but have not been tested in in vivo retrovirus infection models.

**Fig. 2:**
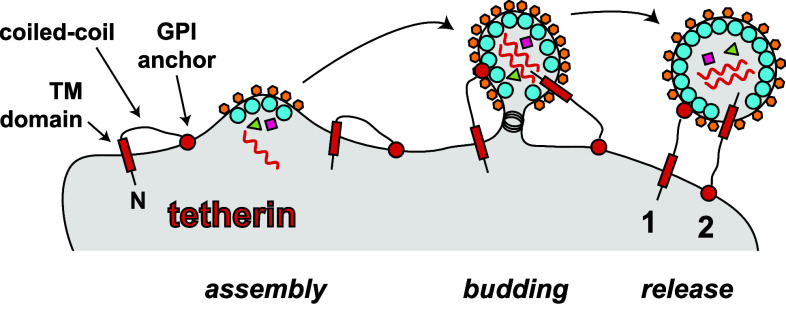
Tetherin prevents release of virus particles from infected cells. Tetherin is shown in *black with red filling*. Tetherin is a type II transmembrane protein. The N-terminus is indicated (*N*) and bears a short cytoplasmic tail, followed by a transmembrane domain (TM), a coiled-coil region and a C-terminal glycophosphatidylinositol (GPI) anchor (*circle*). During budding, either the TM domain or the GPI anchor can be incorporated into virions, resulting in their “tethering” to the plasma membrane. The two different conformations by which tetherin can connect the cell membrane with the viral envelope are indicated (*1 and 2*). Please note that tetherin forms a dimer via coiled-coil interactions, which is not shown here for simplicity

The most intensely studied host protein restricting HIV-1 at the membrane is tetherin (also known as BST-2, CD317, HM1.24 and PDCA-1). Its function is antagonised by the HIV-1 accessory protein Vpu. In fact, the observation that Vpu-deficient viruses are released inefficiently from infected cells led to the discovery of tetherin as a restriction factor in 2008 [34, 35]. Tetherin is a dimeric type II transmembrane protein bearing a short N-terminal cytoplasmic tail, followed by a transmembrane domain, a long α-helical region and a C-terminal glycophosphatidylinositol (GPI) membrane anchor [36]. As such, tetherin inserts into membranes twice with both its N- and C-terminus, and this explains its antiviral activity: tetherin connects or “tethers” the cell membrane to the envelope of newly budded virus particles and this prevents virus release from the cell [34–36]. Tetherin can bridge between the cell and the virus in two different configurations: the transmembrane domain can be inserted either into the cell membrane or into the viral envelope (Fig. 2). In the former case, the GPI anchor is inserted into the virus, whereas in the latter case, it is found in the cell membrane [36]. HIV-1 Vpu antagonises tetherin by interfering with its transport to the plasma membrane and by re-routing tetherin to a degradative compartment [36]. Some other lentiviruses have evolved alternative strategies to counteract tetherin involving the Env and Nef proteins [36]. Whilst strong in vitro evidence shows that tetherin interferes with the release of free virus from infected cells into the surrounding culture medium, the protein may actually enhance cell-to-cell spread of HIV-1 [37]. This perhaps occurs by concentrating virus at the surface of an infected cell [37], although this notion is controversial [38, 39].

Two studies tested the in vivo function of tetherin using independent lines of knockout mice [40, 41]. Tetherin-deficient mice are viable and have no easily recognisable phenotype. Moreover, the development of lymphocytes and other cells of the immune system appears to be unaffected in the absence of tetherin [40, 41]. In one study, infection of wild-type and tetherin knockout mice with exogenous Moloney murine leukaemia virus (M-MLV) resulted in identical virus replication and pathogenesis [40]. In the other study, splenic M-MLV titres 11 days after infection were slightly higher in tetherin-deficient mice than those in wild-type control animals, although the difference was not statistically significant [41]. Both human and mouse tetherin are encoded by type I interferon stimulated genes [36, 40]. As such, tetherin expression is low at baseline. Moreover, in vivo M-MLV infection does not induce appreciable amounts of type I interferon and does not induce tetherin expression, providing an explanation for the lack of a clear phenotype in infected tetherin knockout mice [40, 41]. Consistently, when type I interferons—and as a consequence tetherin—are experimentally induced in vivo by administration of poly I:C during an established M-MLV infection, viral titres are reduced in wild-type mice but not in tetherin-deficient animals [40]. Moreover, another strain of MLV (LP-BM5), which naturally induces type I interferons, replicated to higher titres and caused increased pathology in tetherin knockout mice [40]. Taken together, these data from knockout mice suggest that tetherin controls retrovirus infection in vivo—by limiting virus levels and pathogenesis—as long as its expression is induced by type I interferons. Consistent with this notion, in vivo depletion of tetherin by local administration of siRNAs enhances MMTV replication [42]. Interestingly, a tetherin polymorphism naturally occurring in NZW/LacJ mice truncates the N-terminal cytosolic tail of the protein [43]. As a result of this truncation, cell surface expression of tetherin is increased compared to C57BL/6 mice that express canonical tetherin [43]. In a Friend virus infection model, virus replication and pathogenesis inversely correlated with tetherin cell surface levels [43], underscoring that tetherin is a retrovirus restriction factor not only in vitro but also in vivo.

Although tetherin was initially described as an HIV-1 restriction factor and has been primarily studied in the context of retrovirus infection, its mechanism (retaining viral particles at the cell surface by inserting into the viral envelope) suggests that tetherin might also target other enveloped viruses. Indeed, tetherin limits release of herpesviruses [44, 45], filoviruses [46] and flaviviruses [47, 48]. In addition, tetherin has been suggested to control influenza A virus [49–52], although some studies do not agree with this conclusion [53, 54]. The availability of tetherin-deficient mice opens the exciting possibility to test if and how this restriction factor controls non-retroviral pathogens. Surprisingly, in an initial set of experiments, Colonna and colleagues found that while in vivo replication of mouse cytomegalovirus (a herpesvirus) was comparable in wild-type and tetherin-deficient mice, titres of influenza B virus (an orthomyxovirus) and vesicular stomatitis virus (a rhabdovirus) were actually reduced in knockout mice [41]. A possible explanation is that tetherin may have a function in cellular uptake of these viruses. Consistent with this notion, in the case of human cytomegalovirus, tetherin appears to be advantageous to the virus by enhancing entry into target cells [55]. Other reasons for the decreased replication of some viruses in tetherin-deficient mice may relate to a possible role of tetherin in type I interferon production by plasmacytoid dendritic cells and/or in the adaptive immune response [41]. Tetherin gene-targeted mice will be key tools to study these questions. Another important issue that should be addressed in tetherin knockout mice is its role as a signalling molecule activating NFκB [36]. Indeed, in vitro studies suggest that tetherin acts as a virus sensor and induces the expression of proinflammatory genes via NFκB [56–58], but the impact this has in vivo is unknown.

## Intracellular HIV-1 restriction before integration

### SAMHD1 restricts reverse transcription

After entry of the virus into the cytosol, reverse transcription of the retroviral RNA genome occurs. The recently identified restriction factor SAMHD1 targets this step in the life cycle of HIV-1 [59–61]. The discovery of SAMHD1 as a restriction factor goes back to the observation that HIV-1 infection is inefficient in quiescent myeloid cells such as dendritic cells [62–64]. The closely related virus HIV-2 infects these types of cells much more readily. This difference between HIV-1 and HIV-2 has been attributed to Vpx, an accessory protein encoded by HIV-2 but not by HIV-1. Indeed, infection of myeloid cells with HIV-1 is markedly enhanced in the presence of Vpx, which overcomes a post-entry block prior to or at the level of reverse transcription [64–66]. At the time, this suggested that myeloid cells express a restriction factor and that Vpx inactivates this protein, which perhaps interferes with reverse transcription.

Two proteomic studies identified SAMHD1 as a host protein co-purifying with Vpx [67, 68]. These studies also showed that Vpx recruits a cellular ubiquitin ligase complex to SAMHD1, targeting the protein for proteasomal degradation [67, 68]. As a result, SAMHD1 protein levels are greatly reduced in the presence of Vpx [67, 68]. Importantly, depletion of SAMHD1 in myeloid cells by RNA interference at least partly phenocopies the effect of Vpx and facilitates infection with HIV-1-derived lentivectors [67, 68]. Taken together, these two landmark papers published in 2011 identified SAMHD1 as a new HIV-1 restriction factor [67, 68]. Since then rapid progress has been made in characterising in detail how SAMHD1 controls infection [59–61].

**Fig. 3:**
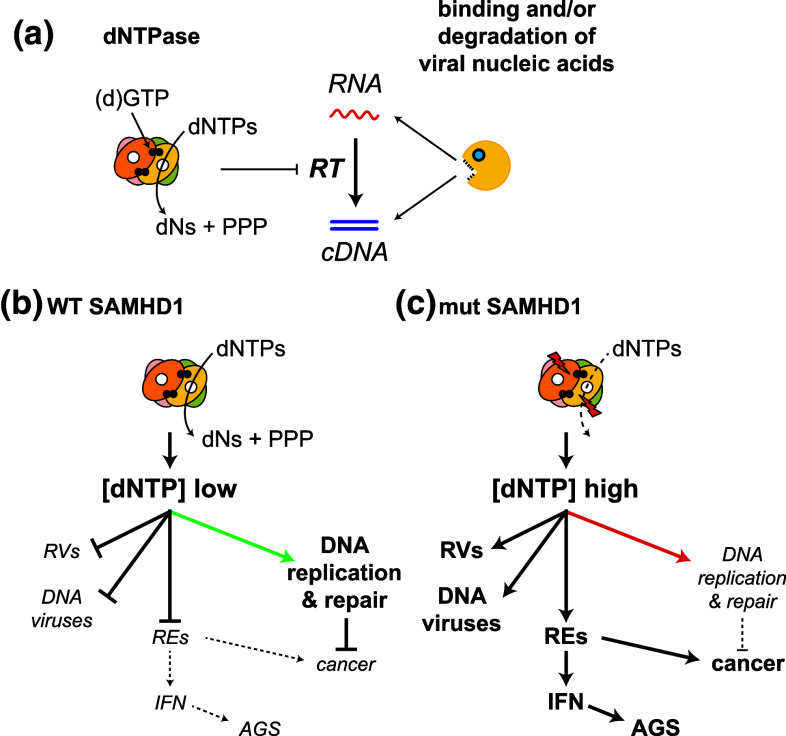
Degradation of dNTPs by SAMHD1 controls reverse transcription and may impact on other cellular processes. **a** Two alternative mechanisms by which SAMHD1 may inhibit reverse transcription (RT) are indicated. (*Left*) SAMHD1 forms a tetramer [85, 86, 192] that cleaves dNTPs—the substrates required for RT—into deoxyribonucleosides (dNs) and inorganic triphosphate (PPP). Each monomer has an active site (*white circle*) and dGTP (or GTP [192, 193]; *black circles*) is bound at an allosteric site. (*Right*) SAMHD1 may also counteract reverse transcription by binding and/or degrading viral nucleic acids. **b** By lowering the intracellular dNTP concentration ([dNTP] low), SAMHD1 not only restricts retroviruses (RVs) but also DNA viruses and perhaps endogenous retroelements (REs). Moreover, balanced dNTP pools are likely to be required for accurate DNA replication and DNA repair (*green arrow*). **c** Mutations in SAMHD1, which have been described in Aicardi-Goutières syndrome (AGS) and cancers such as chronic lymphocytic leukaemia, are predicted to disrupt the proteins’ catalytic function. As a result, dNTP concentrations in cells are elevated ([dNTP] high). This facilitates replication of retroviruses (RVs), DNA viruses and possibly retroelements (REs). Detection of REs by the innate immune system could result in chronic production of type I interferons (IFN) triggering the onset of AGS [194]. Insertion of REs into new positions in the genome could also be a source of mutations leading to the development of cancer. Moreover, imbalanced dNTP concentrations in the absence of functional SAMHD1 may impact on the fidelity of DNA replication and repair (*red arrow*) or on cell cycle progression, further promoting genome instability and transformation [195, 196]

SAMHD1 has two protein domains: an N-terminal sterile alpha motif (SAM) and a central HD-domain. A variety of proteins comprise either a SAM- or an HD-domain, but SAMHD1 is the only protein described to this date in which these two domains are found together. HD-domains found in other proteins often mediate phosphohydrolase activities [69]. This led to the discovery that SAMHD1 degrades deoxynucleoside triphosphates (dNTPs), the building blocks of DNA [70, 71]. SAMHD1 cleaves all four dNTPs, releasing inorganic triphosphate and nucleosides [70, 71]. Consistent with these observations, depletion of SAMHD1 from cells by Vpx delivery, RNA interference or genetic ablation results in elevated intracellular dNTP concentrations [72–77]. This suggests that SAMHD1 restricts HIV-1 by degrading dNTPs, the substrates used by reverse transcriptase during cDNA synthesis (Fig. 3a) [78]. This mechanism has been variably called “nucleotide embargo” or “nucleotide starvation” [60, 79]. Indeed, dNTP concentrations in human macrophages—a cell type in which SAMHD1 restriction is very efficient—are lower than the K_M_ of HIV-1 reverse transcriptase for dNTPs [72, 80, 81]. Importantly, dNTP levels are increased above the K_M_ after SAMHD1-depletion in human macrophages [72, 74].

In addition to its function as a dNTP hydrolase, SAMHD1 has been described to bind to nucleic acids [82, 83] and to have nuclease activity [84]. Currently available structures of SAMHD1 neither reveal the presence of an RNA or DNA binding domain nor of a nuclease domain [70, 85, 86]. However, the N-terminus of SAMHD1 containing the SAM domain is lacking from these crystal structures and some SAM domains are protein-RNA interaction modules [87]. It is, therefore, plausible that full-length SAMHD1 binds nucleic acids and perhaps has nuclease activity. This may explain why some studies find that mutant forms of SAMHD1, which have normal dNTPase activity, fail to restrict HIV-1 in in vitro overexpression settings [88, 89] and that the effect of Vpx cannot entirely be recapitulated by provision of cells with an excess of deoxyribonucleosides [90]. As such, sequestration and/or degradation of HIV-1-derived nucleic acids and “dNTP starvation” are alternative—although not necessarily mutually exclusive—mechanistic explanations for SAMHD1’s activity blocking reverse transcription (Fig. 3a).

Two studies published in 2013, one by the Roers group and one by our laboratories, described independent lines of SAMHD1 knockout mice [76, 77]. These animals develop normally, are fertile and do not display any obvious abnormalities. Both studies show that dNTP levels are increased in SAMHD1 knockout cells, providing genetic evidence that dNTP hydrolysis is an evolutionarily conserved function of SAMHD1 [76, 77].

Experiments in which these mice were challenged with HIV-1 based lentivectors yielded some interesting insights into the biology of SAMHD1 [91, 92]. We started by infecting bone marrow derived dendritic cells and macrophages, as well as live mice, with VSV-G pseudotyped lentivectors [76]. Unexpectedly, infection levels in wild-type and SAMHD1-deficient cells in vitro and in mice in vivo were identical [76], in contrast to what had been observed in human cells depleted of SAMHD1 and infected with the same or very similar lentivectors [67, 68, 72]. Surprisingly, we found the dTTP concentration in mouse dendritic cells to be 0.5 μM [76], while human monocyte-derived macrophages contain around 0.05 μM dTTP [72, 80, 81]. Whether this reflects a species difference between humans and mice or is simply due to differences in cell type and/or culture conditions remains to be seen. The K_M_ of HIV-1 RT for dTTP is approximately 0.07 μM [80]. Therefore, a possible explanation for the lack of SAMHD1-dependent restriction in mouse dendritic cells is that dNTP pools are not limiting reverse transcription. To test this idea, we infected cells and mice with a reverse transcriptase point mutant (V148I) lentivector [76]. This mutation increases the K_M_ of reverse transcriptase for dNTPs [93]. The infectivity of lentivectors with this mutant reverse transcriptase was reduced in wild-type cells and mice [76]. This restriction was relieved by SAMHD1-deficiency, both in vitro in different types of cells as well as in vivo [76]. Taken together, these results show that SAMHD1 not only controls infection in cultured cells but also in a living organism. Similar conclusions were drawn from infection experiments performed in the other strain of SAMHD1 knockout mice [77]. Some—but not all—of the lentivectors used in this study were controlled by SAMHD1 in the presence of a wild-type reverse transcriptase [77]. Much like what was discussed earlier for tetherin and MLV infection, these differences perhaps relate to type I interferons that enhance SAMHD1 expression [76, 94]. Type I interferons are only induced by some lentivectors: second-generation vectors with a minimal genome do not induce type I interferons [64, 76], while first-generation vectors with a full genome do [64] [our own observations].

The observation that viruses bearing a polymerase with lower binding to nucleotides become increasingly sensitive to SAMHD1 strongly suggests that “dNTP starvation” is key to the mechanism by which SAMHD1 restricts infection. Indeed, the reverse transcriptase V148I mutant virus was also attenuated in human cells, and this attenuation was partly relieved by Vpx-mediated SAMHD1 degradation [72]. Moreover, SAMHD1 facilitates apoptosis in monocytes that is induced by infection with the delta-retrovirus human T cell leukaemia virus type 1 [95]. This effect can be reversed by provision of deoxyribonucleosides [95], again pointing to hydrolysis of dNTPs as the mechanism of SAMHD1’s antiviral activity. In short-term in vivo infection experiments using two different mouse gamma-retroviruses, Friend virus and M-MLV, virus replication and virus-induced pathology were comparable in wild-type and SAMHD1-deficient mice [76, 77]. Friend virus and M-MLV replicate in actively dividing lymphocytes. These cells contain large amounts of dNTPs needed to support genomic DNA replication. As such, the lack of SAMHD1-dependent restriction is likely due to dNTPs not being limiting for reverse transcription. In addition, posttranslational modification of SAMHD1 in cycling cells by phosphorylation has been suggested to inactivate its function to repress reverse transcription [88, 89, 96].

Our comparison of lentivectors bearing a wild-type and a mutant reverse transcriptase with reduced binding to dNTPs, and the finding that only the latter virus is potently controlled by SAMHD1 in vivo [76], also provide an explanation for the absence of a *Vpx* gene from the HIV-1 genome: the high affinity of the HIV-1 reverse transcriptase for dNTPs may partially negate SAMHD1’s role in reducing intracellular dNTP concentrations. Indeed, although SAMHD1 potently restricts HIV-1 in cultured human cells, this effect is not absolute: typically, a small fraction of cells is infected in the presence of SAMHD1 [67, 68, 72]. Interestingly, HIV-2’s reverse transcriptase is less efficient than HIV-1’s enzyme at low dNTP concentrations [97]. It, therefore, appears that these two closely related retroviruses use very different strategies: HIV-2 encodes Vpx that targets SAMHD1 for degradation by the proteasome, while HIV-1 evolved a reverse transcriptase with a low K_M_ for dNTPs to partially evade the function of SAMHD1.

In this context, it is interesting to speculate that some degree of control exerted by SAMHD1 might actually be advantageous for HIV-1 at the level of an infected host—by virtue of restricting the virus at the cellular level. Infection of dendritic cells with viruses, including HIV-2, is often a strong signal for dendritic cell activation [64, 98]. However, this is not the case during HIV-1 infection [64]. Degradation of SAMHD1 via experimental Vpx delivery results in dendritic cell activation during HIV-1 infection and allows these cells to prime a T cell response [64]. In this setting, reverse transcribed cDNA is recognised by the recently identified cytosolic DNA sensor cGAS [99, 100] (for a review of the cGAS pathway, see [101]). In the presence of SAMHD1, the amount of reverse transcription products made in dendritic cells during HIV-1 infection is reduced due to low dNTP concentrations and possibly other mechanisms. This results in reduced cGAS stimulation, dendritic cell activation and T-cell priming [100]. This is perhaps beneficial to HIV-1 and might explain why HIV-1 is more pathogenic than HIV-2 [100, 102]. Notably, the concept that cellular factors, which prevent the accumulation of reverse transcribed cDNA, are advantageous for the virus in some settings by preventing the induction of an immune response had been proposed earlier in the context of TREX1 [103].

SAMHD1 is a conserved protein and orthologues have been identified in a variety of organisms, from marine invertebrates up to man [104]. This suggests that the evolutionarily ancestral function of SAMHD1 may not be related to the control of lentiviruses. Indeed, several additional functions of SAMHD1 have been suggested (Fig. 3b, c):

#### Control of DNA viruses

Recent studies show that SAMHD1 also restricts DNA viruses, including herpesviruses and poxviruses [105, 106]. A thymidine kinase-deficient vaccinia virus strain was more sensitive to SAMHD1 than the corresponding wild-type virus [106]. Thymidine kinase ensures the supply of dNTPs for viral genome replication. It is tempting to speculate that this viral enzyme has evolved as a SAMHD1 antagonist and it will be interesting to test restriction of DNA viruses in vivo in SAMHD1 knockout mice.

#### Tumour suppression and prevention of autoimmune disease

Mutations in SAMHD1 have been identified in malignant B-cells isolated from chronic lymphocytic leukaemia patients [107–109] and in other types of cancer [110–112]. Indeed, SAMHD1 has been suggested to be a tumour suppressor [109, 113]. *SAMHD1* is also one of the genes linked to Aicardi-Goutières syndrome, a hereditary autoinflammatory disease characterised by spontaneous and chronic production of type I interferons [104, 114]. It is noteworthy that the ability of cells from Aicardi-Goutières syndrome patients with *SAMHD1* mutations to control HIV-1 is impaired [75, 115, 116].

#### Control of retroelements

A significant fraction of the human genome is composed of endogenous retroviruses and retrotransposons, collectively called retroelements, and the same is true for most other sequenced genomes [117]. Two recent studies found that SAMHD1 prevents retro-transposition of retroelement reporter constructs [85, 118]. It is, therefore, possible that SAMHD1 represses at least some retroelements, most likely by controlling their reverse transcription (Fig. 3b, c).

A function of SAMHD1 in suppressing reverse transcription of endogenous retroviruses and retrotransposons may explain the role of SAMHD1 in cancer and autoimmunity (Fig. 3b, c). Activation of retroelements in cells lacking functional SAMHD1 could result in mutations caused by insertions of retroelements into new sites in the genome. Moreover, detection of reverse transcribed cDNA by innate immune sensors may lead to the production of type I interferons and ultimately Aicardi-Goutières syndrome [119]. These are exciting questions for future research that will benefit from the availability of SAMHD1-deficient mice [76, 77, 91, 92].

### APOBEC3 cytidine deaminases

The AID/APOBEC proteins are cytidine deaminases and convert cytosine to uracil in either DNA or RNA molecules. They constitute a family of 11 members in humans and 5 in mice [120, 121]. The family can be divided into five subgroups: AID and APOBEC1-4 [120]. In mice, each subfamily has a single member, while in humans the APOBEC3 subfamily expanded and has 7 members called APOBEC3A-H [120]. The AID/APOBEC family is characterised by the presence of one or two cytidine deaminase-like domains: AID, APOBEC1, -2, -3A/C/H and -4 have one cytidine deaminase-like domain, while APOBEC3B/D/F/G as well as mouse APOBEC3 have two such domains [120]. AID proteins introduce mutations into immunoglobulin genes and thereby contribute to antibody diversification [122]. APOBEC1 edits a specific messenger RNA and changes its coding potential, while little is known about APOBEC2 and -4 [120, 121]. The APOBEC3 subfamily and in some species APOBEC1 are involved in cell-intrinsic antiviral host defence and in the control of retroelements [121, 123]. In particular, APOBEC3G has been studied in detail and can be considered as the first described HIV-1 restriction factor [4, 5, 123]. Several other members of the APOBEC3 subfamily have also been implicated in the restriction of HIV-1. Moreover, APOBEC3 proteins control other viruses including human T-cell leukaemia virus type 1 and hepatitis B virus. These findings have been summarised in a number of excellent review articles (for example, [4, 5, 121, 123–126]) and will not be discussed in detail here.

As was the case for tetherin and SAMHD1, the discovery of APOBEC3G as a restriction factor was helped by studies of an HIV-1 accessory gene: *Vif*. Indeed, the observation that Vif protein was required for virus infectivity in some types of cells but was dispensable in other cells led to the identification of APOBEC3G as a cellular factor controlling HIV-1 in 2002 [127]. Vif-deficient viruses can only replicate in cells that do not express APOBEC3G [127]. Vif targets APOBEC3G for proteasomal degradation and may additionally inhibit APOBEC3G expression or function [4, 5].

**Fig. 4:**
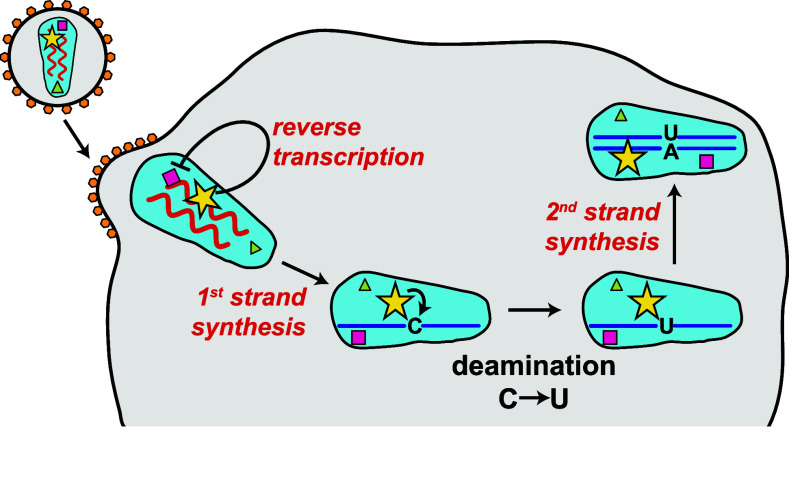
APOBEC3G introduces mutations into viral cDNA and prevents reverse transcription. APOBEC3G (*yellow star*) is incorporated into virus particles during budding and is associated with the viral core. It is, therefore, delivered into newly infected cells. APOBEC3G directly inhibits reverse transcription, for example by blocking the progression of reverse transcriptase (*violet square*). APOBEC3G also deaminates cytosine to uracil in minus-sense single-stranded cDNA, causing extensive incorporation of deoxyadenosine instead of deoxyguanosine during plus-strand synthesis. This introduces mutations into the viral genome that can be deleterious, a process called hypermutation

What is the mechanism by which APOBEC3G counteracts HIV-1? APOBEC3G is packaged into virus particles, particularly in the absence of Vif [4, 5]. As such, APOBEC3G is delivered into newly infected cells. APOBEC3G is associated with the viral core containing the viral RNA genome [4, 5]. This allows the protein to gain access to newly reverse transcribed cDNA. APOBEC3G converts cytosine to uracil in minus-sense single-stranded cDNA, causing extensive incorporation of deoxyadenosine instead of deoxyguanosine during plus-strand synthesis, which results in hypermutation of the viral genome [4, 5]. These G-to-A mutations in viral cDNA are believed to be deleterious to the virus [4, 5]. APOBEC3G also counteracts HIV-1 by additional mechanisms, for example by blocking translocation of reverse transcriptase (Fig. 4) [4, 5].

An antiviral role of APOBEC3G and related proteins in humans is supported by studies of virus hypermutation rates, APOBEC3 expression levels and sequence polymorphisms, and from analysis of how these correlate with clinical parameters [128]. Here, I will summarise insights from experiments in mice, which have a single *Apobec3* gene. Remarkably, the existence of a gene in mice called *Rfv3* that contributes to the immune response to Friend virus, particularly to virus-specific antibody production, had been noticed in the late 1970s [129, 130]. 30 years later, genetic mapping indicated that *Rfv3* could correspond to the *Apobec3* gene [131], which was then confirmed by genetic inactivation of *Apobec3* in an *Rfv3*-resistant strain [132]. Alternative splicing of APOBEC3 pre-messenger RNA causes a Friend virus susceptible phenotype in some mouse strains [132, 133], suggestive of an important role for APOBEC3 in controlling retrovirus infection. Like human APOBEC3G, mouse APOBEC3 is packaged into retrovirus particles, including MMTV and MLV virions [134–136]. Mouse APOBEC3 can also incorporate into HIV-1 particles when it is overexpressed in human producer cells, although this is not antagonised by Vif [137].

Several studies tested the role of APOBEC3 in vivo by genetic ablation in the mouse [132, 134, 138, 139]. In one, a neomycin cassette was inserted into the *Apobec3* locus by conventional gene targeting [138]. Three other studies generated APOBEC3-deficient animals using the same embryonic stem cell line, in which the *Apobec3* gene had been disrupted by gene-trap technology [132, 134, 139]. APOBEC3-deficient mice develop normally and have no apparent phenotype [132, 134, 138]. Initial experiments, in which wild-type and knockout mice were challenged with retroviruses, demonstrated that APOBEC3 acts as a restriction factor in vivo. Indeed, MMTV replicates and spreads better in APOBEC3-deficient mice [134, 140]. Similarly, gamma-retroviruses, including Friend virus, M-MLV and LP-BM5, are controlled by APOBEC3 in vivo: these viruses replicate to higher titres in knockout mice and infection results in increased pathogenesis [132, 141–143]. These studies also revealed that not only virion-packaged APOBEC3 but also APOBEC3 expressed by the target cell contributes to virus restriction [142]. Furthermore, restriction of exogenous retroviruses by mouse APOBEC3 appears to be largely independent of deamination [134, 135], despite the observation that the recombinant protein possesses cytidine deaminase activity [136]. Instead, APOBEC3 restricts infection before or at the step of reverse transcription, although the exact mechanism remains to be determined [144, 145]. The deaminase activity of APOBEC3 plays perhaps a more important role in controlling endogenous retroviruses [146]. In contrast to the results obtained with retroviruses, in vivo replication of mouse gammaherpesvirus 68 is unaltered in APOBEC3 knockout mice [139].

A series of further studies using APOBEC3-deficient mice revealed a number of interesting insights into the role of this restriction factor in controlling retrovirus infection in a living organism. In one set of experiments, the role of APOBEC3 in virus transmission was tested. Both vertical transmission of MMTV via milk and horizontal, sexual transmission of LP-BM5 are controlled by APOBEC3 [143, 147]. In another set of studies, the impact of APOBEC3 on the development of an adaptive immune response during Friend virus infection was dissected. APOBEC3 was found to be required for the production of neutralising antibodies in vivo [132, 148, 149]. These data have been reviewed in detail elsewhere [150] and may be of relevance to the development of vaccines. An interesting mechanism of viral APOBEC3 inhibition was also revealed in the knockout mice. Many strains of MLV express a glycosylated form of the viral gag protein called glycogag. Glycogag mutant viruses are attenuated in wild-type, but not in APOBEC3 knockout mice [151, 152]. Glycogag forms part of the virus capsid surrounding the viral genome and is required for capsid stability [151]. Glycogag may, therefore, prevent access of APOBEC3 and perhaps other cellular proteins to reverse transcribed cDNA [151]. This mechanism is distinct from the APOBEC3G antagonism exerted by HIV-1 Vif that prevents packaging of the restriction factor into virus particles [5]. It will be interesting to test whether the HIV-1 capsid has evolved to interfere with APOBEC3 proteins. It is noteworthy that the HIV capsid limits access of other host factors to viral nucleic acids and thereby prevents their detection by innate immune sensors, particularly cGAS [100, 153].

Several restriction factors are induced by type I interferons and proinflammatory stimuli such as lipopolysaccharide (LPS). This includes the human APOBEC3 proteins [5] and also mouse APOBEC3, although in the latter case the level of induction by type I interferons appears to be weak [94, 154]. Administration of LPS or type I interferons blocks the replication of many viruses in vivo. Interestingly, the negative effect of LPS on MMTV replication in wild-type mice was largely cancelled in APOBEC3-deficient animals [154]. Similarly, type I interferons failed to control Friend virus in APOBEC3 knockout mice [94]. A possible interpretation of these results is that APOBEC3 plays a particularly important and non-redundant role in in vivo control of these viruses [94, 154]. Indeed, a hierarchy of restriction factors had been suggested based on experiments using mice with higher cell surface expression of tetherin due to a polymorphism [43]. Interestingly, the effect of this polymorphism to decrease Friend virus replication can only be revealed in mice that have an alternatively spliced form of APOBEC3 linked to Friend virus susceptibility [43]. In the presence of the canonical *Apobec3* allele that confers resistance to Friend virus, lower tetherin expression levels did not relieve in vivo restriction, pointing to a degree of redundancy amongst restriction factors [43]. It will be interesting to test the virus susceptibility of mice lacking more than one restriction factor to determine how these proteins work together in vivo. The availability of tetherin-, SAMHD1- and APOBEC3-deficient animals opens the possibility of generating double and perhaps triple knockout mice. Moreover, it may be worthwhile to cross breed transgenic mice that express human proteins required for cellular entry and replication of HIV-1 (such as human CD4) [155] with animals that lack one or multiple restriction factors. It is exciting to speculate that such mouse models will perhaps recapitulate some steps of the HIV-1 life cycle.

### Other restriction factors acting prior to integration

HIV-1 infection is controlled by a variety of other cellular proteins that can be classified as restriction factors based on their cell-intrinsic activities in blocking infection [156]. This includes several members of the TRIM protein family, MOV10 and Mx2. These restriction factors will be discussed only briefly here, given the lack of in-depth in vivo studies in mouse knockout models.

TRIM5α has been characterised in detail and targets the capsid of HIV [5, 157]. TRIM5α lacks a clear orthologue in mice (and as such will not be discussed further in this review), but it has been proposed that the Friend virus restriction factor Fv1 is similarly directed at retroviral capsids [14]. Other TRIM proteins are also involved in the control of exogenous retroviruses and endogenous retroelements, particularly in limiting transcription of integrated proviral DNA [158]. Examples include TRIM28 (also known as KAP1) and TRIM24, and knockout mouse cells revealed a role of both proteins in controlling endogenous retroviruses [159, 160].

MOV10 and its paralogue MOV10L1 are helicase proteins [161]. In vitro, MOV10 overexpression in HIV-1 or MLV producer cells results in reduced virus titres [162–165]. As is the case for APOBEC3G, MOV10 is packaged into HIV-1 particles and, upon delivery into newly infected cells, inhibits infection by a mechanism that perhaps targets reverse transcription [163–166]. Interestingly, MOV10 also controls endogenous retroelements [162, 167, 168]. MOV10 knockout mice have not been published; however, MOV10L1 has been targeted and mice lacking this helicase have a defect in controlling retrotransposons [169, 170]. This defect results in male infertility and a complete block of spermatogenesis due to increased activity of retrotransposons in the male germ line [169, 170]. Mechanistically, MOV10L1 has been suggested to be involved in the piRNA pathway that controls retrotransposons [169–171].

Mx2 (also known as MxB) is a recently identified HIV-1 restriction factor in human cells [172–175]. Mx2 is induced by type I interferons and is related to Mx1, an important restriction factor of influenza A virus [176]. Although the details of the mechanism by which Mx2 restricts HIV-1 are not fully understood, it appears that Mx2 targets a late post-entry step, perhaps disrupting nuclear import of the reverse transcription complex [172–175]. Overexpression of human Mx2 in human cells has no or weak effects on MLV infection [172, 173]. Whether mouse Mx2 controls retroviruses has not been tested. Commonly used inbred strains of mice lack functional Mx2 due to a mutation [177]. I will be interesting to repair this mutation or to introduce a functional copy of the Mx2 gene [178] and to challenge such animals with retroviruses.

## Intracellular HIV-1 restriction after integration

Restriction factors exerting intracellular control of HIV-1 after integration have not been studied in comparable detail as those restriction factors antagonising the virus before integration or at the membrane. Nevertheless, several host proteins targeting HIV-1 transcripts or the translation of viral proteins have been identified and will be briefly mentioned here.

RNase L was one of the first antiviral proteins to be discovered [179]. In the absence of virus infection, RNase L is monomeric and inactive. Delivery of double-stranded RNA into cells during virus infection activates oligoadenylate synthase, which produces 2′-5′ oligoadenylates (2-5A). 2-5A binds RNase L resulting in dimerization and activation of its endonuclease activity. RNase L then degrades viral and host RNAs and thereby mediates an antiviral effect [179]. Several lines of in vitro data suggest that RNase L controls HIV-1 and Friend virus, perhaps by degrading viral transcripts [180–183]. Nevertheless, infection of RNase L knockout mice with Friend virus revealed that this factor is not required for the control of virus levels and the induction of adaptive immune responses [183]. These observations highlight that retrovirus restriction in vitro does not always translate into virus control in an infected host and provide an example for the importance of in vivo studies.

ZAP (also known as ZC3HAV1) is a zinc finger protein that recognises retroviral RNA transcripts and targets them for degradation via the RNA exosome [184–186]. ZAP-deficient mice have not been studied in retrovirus infection settings, but knockout cells support increased in vitro MLV replication suggesting that this protein is a retrovirus restriction factor [187].

Another restriction factor acting after integration is SLFN11 [188, 189], a member of the Schlafen family of proteins that comprises 5 members in human and 10 in the mouse [190]. The expression of these proteins is induced by type I interferons and some family members have a described function in T-cells [190]. Overexpression of SLFN11 in virus producer cells results in lower HIV-1 titres and this correlates with reduced levels of viral proteins made by the producer cell [188]. Conversely, depletion of this protein by RNA interference enhances viral titres and viral protein expression [188]. Interestingly, viral RNA levels are unaffected by SLFN11. Instead, SLFN11 prevents viral protein expression at the level of translation. HIV-1 messenger RNAs have a bias in the use synonymous codons and often have A or U in the third position. This codon usage is suboptimal in mammalian cells due to the low abundance of cognate transfer RNAs. As such, viral messenger RNAs are translated inefficiently, and it appears that SLFN11 further decreases their translation. The precise mechanism is not fully understood, but it relates to the ability of SLFN11 to bind transfer RNAs [188]. This is predicted to further limit the availability of rare transfer RNAs, perhaps by sequestration or more indirectly by an impact on transfer RNA maturation or aminoacylation/deacylation [188]. The absence of a clear 1-to-1 orthologue of human SLFN11 in the mouse precludes direct knockout studies. The diversification of this protein family in different species might be related to a role in host defence against evolving pathogens, and further studies into antiviral functions of these proteins are justified.

## Conclusions and perspectives

Mouse knockout models have in several instances provided in vivo evidence for the antiviral activities of restriction factors. Valuable insights into the cell-intrinsic function and mechanism of action of virus restriction factors have been gained from studies in these animals or in cells derived from them. Moreover, mouse models led to interesting insights into the biology of restriction factors in the context of virus infection—such as their impact on adaptive immune responses—and into their role in other settings such as autoimmunity. Therefore, it will be important to gene target more recently identified restriction factors that have not been studied in vivo yet (Table 1). It will also be interesting to generate new mouse lines that lack multiple restriction factors, or a restriction factor and another protein involved in the immune response to virus infection (such as a pattern recognition receptor) to test how these proteins work together in vivo.

Gene targeting has until recently been largely limited to the mouse that will certainly remain an important model in the years to come. New genome editing tools have become available in the last few years, including one based on the bacterial CRISPR-Cas9 system [1]. An exciting development is the possibility to genetically modify monkeys with these tools [191]. As such, it is at least theoretically possible to generate knockout primates that lack a restriction factor. Such a model may provide the means to study lentivirus control by restriction factors in their natural hosts.
